# Social Contagion in COVID-19 Discussions Within the Belgian Reddit Community: Statistical and Modeling Study

**DOI:** 10.2196/87723

**Published:** 2026-07-29

**Authors:** Tim Van Wesemael, Luis Enrique Correa Rocha, Tijs W Alleman, Jan M Baetens

**Affiliations:** 1 BionamiX, Department of Data Analysis and Mathematical Modelling, Faculty of Bioscience Engineering, Ghent University, Coupure Links 653, Ghent, Flanders, 9000, Belgium, 32 9 264 59 32; 2 Department of Economics, Faculty of Economics and Business Administration, Ghent University, Ghent, Flanders, Belgium; 3 Department of Physics, Faculty of Science, Ghent University, Ghent, Flanders, Belgium; 4 Department of Public and Ecosystem Health, College of Veterinary Medicine, Cornell University, Ithaca, NY, United States; 5 Department of International Health, Bloomberg School of Public Health, Johns Hopkins University, Baltimore, MD, United States

**Keywords:** COVID-19 mitigation, Reddit, topic modeling, sentiment analysis, bounded-confidence model, social contagion

## Abstract

**Background:**

Understanding how sentiment toward COVID-19 mitigation measures evolves on social networks can help to inform infectious disease models and policymakers. Even though numerous studies have described social media interactions during the pandemic, few have modeled the underlying dynamics of sentiment contagion and polarization.

**Objective:**

This study aimed to investigate topic emergence and sentiment evolution in COVID-19 mitigation discussions on r/Belgium, focusing on (1) whether discussion topics exhibited social contagion, (2) whether expressed sentiment displayed homophily, and (3) how this homophily formation can be captured by a mechanistic model.

**Methods:**

We classified posts created on r/Belgium between January 1, 2020, and June 30, 2022, into lockdowns, masks, and vaccination, using a pretrained bidirectional encoder representations from transformers (BERT) topic model, and assigned English posts a sentiment, using a robustly optimized BERT pretraining approach (RoBERTa)-based sentiment classifier. We then examined temporal patterns of post volume and tested for social contagion in topic initiation. Sentiment homophily was quantified by comparing observed comment-parent sentiment pairs to null distributions. The novel smooth latent-expressed bounded confidence (SLEBC) model dynamically captured sentiment evolution, distinguishing between latent sentiment trajectories and noisy expressed sentiment. We tested the model against 2 alternatives, one with a linear update and one without the latent state, using the Watanabe-Akaike information criterion.

**Results:**

Analysis of 655,642 posts made by 28,559 users revealed that post volume was associated with external events such as policy announcements and media reports. There was no evidence of within-Reddit social contagion in topic initiation. However, sentiment exhibited significant homophily, with comment sentiment correlating with parent comment sentiment. The SLEBC model reproduced observed sentiment patterns (Watanabe-Akaike information criterion: –28.5 to –18.4 across topics), outperforming both alternatives (–21.1 to –17.4 for the linear model and 6.8 to 692 for the one without the latent state). It slightly underestimated sentiment homophily but still outperformed the alternatives in this regard. In the SLEBC model, expressed sentiment adapts more strongly to the immediate parent comment than the user’s latent state updates based on their interaction history (proportions of users showing this pattern: 0.74, 0.70, and 0.51 for lockdowns, masks, and vaccination).

**Conclusions:**

Discussion topics on r/Belgium are not associated with social contagion within the platform, but sentiment dynamics are shaped by within-thread interactions. The SLEBC model suggests that users adapt their expressed sentiment to match the post they reply to, highlighting that expressed sentiment may poorly reflect underlying latent sentiment. Infodemic models for Reddit-like platforms could benefit from incorporating external information sources for topic seeding and from using bounded confidence rather than linear contagion mechanisms for sentiment spread.

## Introduction

To mitigate the burden on health care systems during a pandemic, governments may be forced to introduce mandatory measures. The COVID-19 pandemic served as a prime example, as the myriad of mitigation measures had far-reaching societal consequences [1-3]. Initially in 2020, these were mainly nonpharmaceutical interventions, such as lockdowns, contact reductions, and mask mandates [4,5]. As the pandemic progressed, vaccination campaigns were initiated near the end of 2020, and the focus gradually shifted to vaccine distribution during 2021. The success of these interventions depended not only on their epidemiological effectiveness but also on public adherence, which was influenced by individual attitudes, information exposure, and social interactions. Therefore, understanding public sentiment toward these measures is crucial for health informatics, as it can inform epidemiological models, guide public health communication strategies, and support evidence-based policymaking [6,7].

An unprecedented surge of health-related information on social media accompanied the pandemic, leading to what has been termed an “infodemic,” the rapid spread of both accurate and inaccurate information that can hinder effective public health responses [8]. This phenomenon has given rise to infodemiology, the science of monitoring and analyzing digital health information to inform public health practice [9]. Social media platforms have become essential data sources for infodemiology, enabling real-time monitoring of public health sentiment and tracking information diffusion patterns [10]. Research has demonstrated that social media discussions about health topics are influenced by multiple factors. While traditional media coverage and official announcements drive much of the online discourse [11,12], the spread of information within social networks also plays a crucial role. Studies that have established correlations between sentiment expressed online and vaccine uptake highlight the potential of social media data as a proxy for public health attitudes [13,14].

However, infodemics are complex and multifaceted, as sentiment expressed on social media can reflect both support and opposition, and discussions are shaped by misinformation spread [15], echo chambers [16], and platform-specific dynamics. Twitter (now X) has been the most extensively studied platform for COVID-19 sentiment analysis [10,17-19], including research specific to Belgium on monitoring emotions [11] and support for specific mitigation measures [20].

Reddit, with its structured discussion threads and pseudo-anonymous communities, provides a different informative environment for studying online health discussions [21,22]. Unlike Twitter’s brief posts, Reddit enables extended, threaded conversations. Previous research on COVID-19-related subreddits has documented both temporal persistence [23] and shifts [24] in sentiment, regional differences in public concerns [25,26], and the role of external events in driving discussion volume and emotions [12,27,28]. Studies have also shown sentiment homophily, the tendency for users to interact with others with similar sentiment [16]. These patterns can manifest in different forms: pluralism (where individual sentiment is largely independent), consensus (where users converge toward similar sentiment), or polarization (where 2 or more sentiment clusters emerge) [29]. A large-scale experiment on Facebook showed that emotional states can spread through this online social network [30]. While echo chambers have been documented on other platforms, Reddit has shown less pronounced polarization in contexts such as the 2016 US election and vaccination [16,31].

Computational models of social contagion have been applied to understand the mechanisms behind these emergent patterns of sentiment on social platforms [32]. These models distinguish between simple contagion, where a single exposure can trigger behavioral change, and complex contagion, requiring multiple exposures [33,34]. Bounded confidence models, which assume that individuals only interact with others holding sufficiently similar opinions, have been used to reproduce observed steady-state patterns of consensus and polarization on social media [29,35]. In public health, these models have been used to model, for example, the spatial diffusion of vaccine hesitancy [36], or to couple opinion formation with epidemic spread on contact networks [37]. Nevertheless, most studies of online COVID-19 discourse have focused on describing sentiment patterns rather than developing mechanistic models that can explain the underlying dynamics. Here we applied a novel dynamic bounded confidence model to COVID-19 mitigation discussions on Reddit, improving mechanistic understanding of how sentiment evolves in threaded conversations.

This study examined discussions on COVID-19 mitigation measures within the Belgian Reddit community (r/Belgium) from January 1, 2020, to June 30, 2022, focusing on three key topics: lockdowns, masks, and vaccination. Topic modeling and sentiment analysis were used to characterize discussion volume and sentiment dynamics, in pursuit of 3 goals. First, we investigated whether discussion topics exhibited social contagion: a null model for contagion testing revealed no evidence of social contagion in topic initiation. Second, we examined whether expressed sentiment displayed homophily: combining interrupted time series analysis with homophily quantification, we found statistically significant sentiment homophily in the reply structure. Third, we modeled how this homophily formed under local interactions and long-term trends: we developed a novel stochastic bounded confidence model, the smooth latent-expressed bounded confidence (SLEBC) model, that distinguishes between a user’s latent state and the observable sentiment expressed in their comments. The SLEBC model reproduced sentiment distributions and homophily better than two ablations: one that did not distinguish between the two sentiment states, and one that used linear instead of bounded confidence updates.

## Methods

### Data Source and Collection

**Figure 1.**
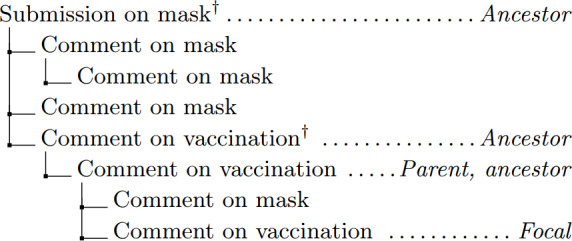
Structure of a Reddit thread. The parent and ancestors of the focal comment are shown. The initiating posts for the mask and vaccination discussions are denoted by †; the others are participating.

Reddit is a social media platform organized into communities called subreddits, denoted by the prefix r/. Users operate under fixed pseudonymous usernames and typically share limited personal information. Discussions begin when a user posts a submission consisting of a title, optionally accompanied by a hyperlink or text. Other users can comment on submissions and reply to specific comments, creating a tree-like conversation structure termed a thread (Figure 1). Throughout this work, the term post refers to both submissions and comments. Each comment’s parent is the post it replies to, and its ancestors comprise the parent and the ancestors of the parent, always including a single submission at the top level. Users can upvote or downvote posts, generating a score reflecting community agreement [21]. A glossary of terms is available in Multimedia Appendix 1.

Posts from the r/Belgium subreddit created between January 1, 2020, and June 30, 2022, were retrieved from the Pushshift dataset [38], a monthly-updated Reddit archive. This period encompassed the phases before, during, and after the COVID-19 outbreak in Belgium. Submissions containing at least one of the keywords listed in Textbox 1 in their title, text, or thread were initially selected. For each post, the unique identifier, creation timestamp, author, text, score, parent post, and submission title were extracted. Markdown tags, URLs, and quotes were removed from all texts.

Textbox 1. **Keywords used to select submissions.**

- corona
- virus
- covid
- mask
- masque
- lockdown
- confin
- quarant
- curfew
- avondklok
- couvre-feu
- couvre feu
- vaccin
- vax
- jab
- booster
- prik
- piqure
- piqûre
- pcr
- plf
- locator form
- cst
- safe ticket

Topic classification was performed using a multilingual bidirectional encoder representations from transformers (mBERT) model, trained specifically for the Belgian context and capable of processing Dutch, French, German, and English text [20]. Although the model identified eight specific measures plus other measures and not applicable categories, analysis focused on three primary measures: lockdowns, masks, and vaccination. These topics were selected for their sustained relevance throughout the pandemic and minimal risk of confusion with non-COVID-19 content. Submissions classified as not applicable with at least 90% of their comments similarly classified were excluded. Comments labeled not applicable or other inherited their parent’s topic. For each topic, the distribution of posts per user was approximated by a power-law distribution $P(k)∼k^{-\gamma }$, where *γ* was determined using maximum likelihood estimation and P(k) is the probability that a user was the author of k posts [39,40].

Language detection was performed using a robustly optimized BERT pretraining approach (RoBERTa), xlm-robertabase-language-detection [41]. For English-language posts, sentiment analysis was conducted with twitter-roberta-base-sentiment-latest (roberta-tbsl) [42], a classifier used for Reddit content [43,44]. The restriction to English avoided language-specific features in sentiment analysis, while incorporating the bulk of the posts (section *Dataset Characteristics*). The roberta-tbsl model outputs three scores (0 to 1) for positive, neutral, and negative tonality. These were combined into a single continuous sentiment value $s_{k}$ per post k by subtracting the negative score from the positive score, yielding values from −1 (most negative) to 1 (most positive). This continuous representation was used in subsequent sentiment analyses (sections *Sentiment Homophily* and *Sentiment Evolution Model*).

### Temporal Analysis

The daily volume of posts related to each topic was calculated and visualized alongside key Belgian policy events, hospitalization counts, and administered vaccination doses [45,46]. A linear interrupted time series model was fitted to daily post counts, using the event dates in Table S1 in Multimedia Appendix 2 as breakpoints [47]. The model assumed a linear segment between consecutive breakpoints. At each event *j*, both the intercept (level change ∆*β*_0*,j*_) and the slope (trend change ∆*β*_1*,j*_) may change, yielding a piecewise linear fit with ordinary least squares CIs. This allowed us to identify changes in posting volume associated with specific events (Multimedia Appendix 3).

To identify days with unusually negative sentiment, each post’s sentiment was weighted by its score and aggregated per day and per topic. A day was marked as a significantly negative day for a topic if the median weighted sentiment on that day fell below the 0.275 quantile of the overall weighted sentiment distribution for that topic and at least 50 comments were submitted that day. These thresholds were chosen to identify 1 to 5 significantly negative days per topic (Multimedia Appendix 3).

### Topic Contagion

To assess whether users were more likely to initiate discussions on a topic after previously participating in one, each post in a thread sharing a common topic was classified as either initiating or participating. A post was classified as initiating if none of its ancestors shared the same topic; otherwise, it was participating (Figure 1). The author of an initiating post was designated the initiator, while all others were participants.

If contagion were present, exposure to a topic through participation would increase the probability of later initiating a new discussion on that topic. Hence, participations would accumulate at the start of a user’s posting sequence. Conversely, if topic initiation is independent of prior participation, initiations and participations should be randomly interleaved across the sequence of a user’s posts.

For each user, the number and sequence of initiations and participations per topic were recorded, along with the position in their posting sequence at which the first initiation occurred. The observed position was compared against a null model that preserves the number of participations and initiations, but treats each possible ordering as equally likely. Under this null model, the probability that the i’th post is the first initiation has a closed-form combinatorial expression (Multimedia Appendix 3). For each user, the observed position in the sequence where the first initiation occurred was compared to the null model distribution. This yielded p(i), the proportion of users with at least one initiation among their first i discussions for a topic, relative to the total number of users that initiated or participated in a discussion on that topic.

### Sentiment Homophily

The relationship between comment and parent sentiment was examined by comparing each comment’s sentiment $s_{k}$ to its parent’s sentiment $s_{l}$. All such pairs $\left(s_{k},s_{l}\right)$ within a topic were collected into a 2D joint histogram with square bins of width $w_{H}=0.05$, yielding an empirical joint probability distribution *H*.

A null model randomly paired comment and parent sentiment by sampling them independently from the observed distributions per topic. For comments, this distribution consisted of observed comment sentiment, while the parent distribution additionally included submission sentiment, making the null histogram $\tilde{H}$ asymmetric. The difference between empirical and null histograms $\Delta H=F_{.05}\left(H-\tilde{H}\right)$, revealed which sentiment pairs occurred more or less often than expected. The function $F_{.05}$ sets entries to zero if their *P* value under the null model exceeds .05. That is, $\Delta H_{l,m}§gt;0$ indicates that the sentiment pair (l,m) occurred significantly more frequently than expected under independent pairing, and $\Delta H_{l,m}<0$ that it occurred significantly less frequently. In the resulting histograms, pluralism appears as uniform patterns, consensus appears as positive mass near a single point on the diagonal, and polarization appears as distinct on-diagonal clusters [29]. This 2D histogram was then summarized into a single homophily measure h(ΔH) by weighting the value of each bin according to its distance from the diagonal l=m. Positive mass near the diagonal increases *h*, while off-diagonal mass decreases it. Higher *h* indicates stronger homophily, while negative values imply heterophily (Multimedia Appendices 3 and 4).

Two contexts served as extensions to comment-parent homophily. In each of them, we include more than one (*n*) preceding comments. First, the ancestral context $A_{k}^{n}$ comprises the n closest ancestors of comment k. Second, the user context $U_{k}^{n}$ includes the parents of the comment k and of the n−1 other comments by the same author on the same topic. If users primarily adapted to recently viewed comments, ancestral homophily would dominate. If users had inherent sentiment and selectively interacted with similar users, user context homophily would prevail.

2D histograms were created for comment sentiment and mean context sentiment for context sizes one through five. Only comments with both ancestral and user contexts of at least size five were analyzed. Homophily values per context size were compared to null histograms ${\tilde{H}}^{n}$ generated by pairing observed sentiment with the mean of *n* random observed sentiments.

### Sentiment Evolution Model

**Figure 2.**
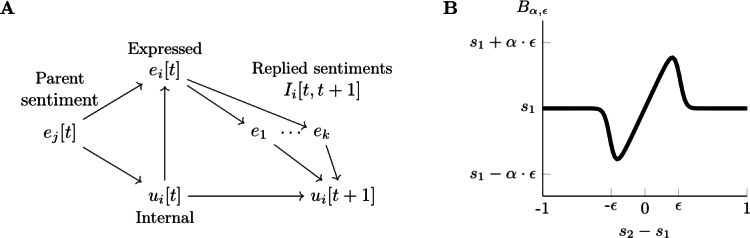
(A) Schematic representation of the smooth latent expressed bounded confidence (SLEBC, Equation 1). (B) The change in sentiment after applying $B_{\alpha ,\epsilon }$ (Equation A.8 in Multimedia Appendix 3) as a function of the sentiment difference $S_{2}-S_{1}$.

The SLEBC model was developed to capture the evolution of a user’s sentiment. The model includes a latent $u_{i}[t]$ and an expressed $e_{i}[t]$ sentiment state for each user i. The former is an unobserved variable that accumulates sentiment influence across all interactions over time, whereas the latter quantifies the sentiment of a specific comment and is observable. This structure is analogous to a state-space model, with *u_i_*[*t*] as the latent state and *e_i_*[*t*] as a parent-influenced observation. The latent state evolves based on the expressed sentiment of other users, that is, the posts that the user replies to, and the comments that the user receives. The expressed state updates according to the user’s latent state and the expressed state of the immediate parent comment, allowing a local adaptation. Both states evolve stochastically according to a smooth bounded confidence kernel $B_{\alpha ,\epsilon }$, each with its own update strength *α* but a shared sentiment distance threshold *ϵ*. Schematically, it is given by Figure 2A, and mathematically, it is given by the following:


(1a) $$e_{i}[t]∼\hat{N}\left(B_{\alpha_{e,i}},\epsilon_{i}(u_{i}[t-1],\;e_{j}[t]),\;\sigma_{e,i}\right)$$

(1b) $$u_{i}[t]∼\hat{N}\left(\underset{e_{k}\in I_{i}[t-1,t]}{⨂}B_{\alpha_{u,i},\epsilon_{i}}(u_{i}[t-1],e_{k}),\;\sigma_{u,i}\right)$$

where t−1 is the time at which user i made its previous comment and $\hat{N}(\mu ,\sigma )$ is a truncated normal distribution with mean *µ* and standard deviation *σ* and support [−1*,*1]. In Equation 1a, $e_{j}[t]$ refers to the expressed sentiment of the user j that user i is reacting to. In Equation 1b, $I_{i}[t-1,t]$ is the set of sentiment of the parent comment and all replies user i received between time t−1 and t, and the operator ⊗ indicates that the bounded confidence update is applied to each such interaction. The user-specific parameters $\alpha_{e,i},\;\alpha_{u,i},\;\epsilon_{i},\;\sigma_{e,i},\;\sigma_{u,i}$ were sampled from a posterior distribution, given the likelihood of the observed expressed sentiments.

In bounded confidence models, interacting agents linearly align their sentiment (with strength α), but only if their difference is smaller than a threshold ϵ, which enables polarized states to emerge [48]. Here, a smoothed version$B_{\alpha ,\epsilon }$, was used to ease calibration (Multimedia Appendix 3) [49]. Figure 2B illustrates the dependence of $B_{\alpha ,\epsilon }$ on the sentiment difference $s_{2}-s_{1}$. Since η is finite, the maximum sentiment update remains smaller than α⋅ϵ. To illustrate the advantage of the bounded confidence kernel $B_{\alpha ,\epsilon }$, it was compared to two alternatives. The first uses a linear kernel to update the sentiment in Equation 1 [50]. This corresponds to the bounded confidence update $B_{\alpha ,\epsilon }$ in Equation A.8 for η→∞ and ϵ=2. Second, we ablated the internal sentiment state, so that expressed sentiment updated directly against the parent comment and received replies, without a separate latent trajectory (Equations A.9 and A.10 in Multimedia Appendix 3).

To fit the SLEBC model and its alternatives, users with at least 40 comments per topic were selected. For each user, the sentiment of their comments and the posts they interacted with was extracted. The posterior distribution of the parameters was obtained using a Hamiltonian Monte Carlo method (Multimedia Appendix 3) [51,52].

To assess the quality of the models, the posterior distribution of predicted sentiment was scored using the Watanabe-Akaike information criterion (WAIC) [53]. A lower value is better and indicates how well the model can predict data it was not trained on (Multimedia Appendix 3). A boxplot synthesizes the homophily values given by h (Equation A.7 in Multimedia Appendix 3), showing their minimum, maximum, and 0.25, 0.5, and 0.75 quantiles. Mann-Whitney *U* tests were used to assess the relative strength of internal and expressed update strengths per user, checking whether the probability $P(\alpha_{e,i}§gt;\alpha_{u,i})$ is larger than $P(\alpha_{e,i}§lt;\alpha_{u,i})$ at the .05 level [54]. The proportion of users for which this is the case is denoted as. Inferred parameters enabled the reconstruction of the latent sentiment trajectory of the users over the period of interest. Monotonic trends in the population median of inferred latent sentiment were identified by a Hamed-Rao test, a modification of the Mann-Kendall test that accounts for autocorrelation [55]. With this test, we checked for each of the subsequent periods delimited by the events in Table S1 in Multimedia Appendix 2 if the population median showed such a monotonic trend with .05 significance.

### Ethical Considerations

This study was not subject to formal ethics board review, as Belgian law restricts such requirements to medical experiments, animal studies, and dual-use research, consistent with Ghent University’s institutional ethics framework [56]. Data were retrieved from the public subreddit, in line with Reddit’s terms of service [57]. Although the raw extract contained identifiers, these were not included in the analytical dataset and were not published; all reported results are aggregated and anonymized. Therefore, this study follows the Association of Internet Researchers ethical guidelines for internet research [58].

## Results

### Dataset Characteristics

The final dataset comprised 28,559 unique users who created 645,280 comments and 10,362 submissions (Table 1). English was the predominant language, accounting for approximately 75% of the posts. Dutch posts represented roughly 10%, while French and German posts were substantially underrepresented, with less than 1% of the posts. Topic distribution was relatively balanced, with lockdowns being most prevalent (94,494 comments, 1009 submissions from 9987 users), followed by masks and vaccination.

**Table 1.** Number of users, comments, and submissions in the considered dataset of posts on r/Belgium (January 2020-June 2022) on COVID-19 mitigation topics.

	Users, n	Comments, n	Submissions, n
Total	28,559	645,280	10,362
Per language			
EN^a^	24,850	488,468	6405
NL^b^	9344	71,940	3240
FR^c^	696	1036	145
DE^d^	272	335	4
Per topic			
Lockdowns	9987	94,494	1009
Masks	6824	48,500	437
Vaccination	5552	41,700	590

a EN: English.

b NL: Dutch.

c FR: French.

d DE: German.

**Figure 3.**
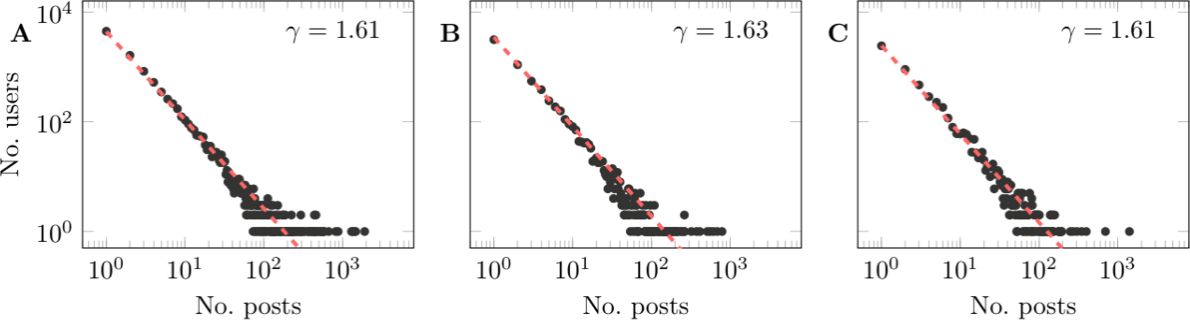
Distribution of posts per user in r/Belgium (January 2020-June 2022) on a double logarithmic scale for (A) lockdowns, (B) masks, and (C) vaccination, and a power-law approximation with exponent γ (dashed, red).

The distribution of posts per user exhibited a heavy-tailed pattern for all topics (Figure 3), consistent with previous observations on Reddit [59]. A small number of users created many posts, while the majority contributed only a single post, limiting the ability to draw conclusions about individual user behavior patterns. Power-law approximations yielded similar exponentsγ for all topics.

### Temporal Analysis

**Figure 4.**
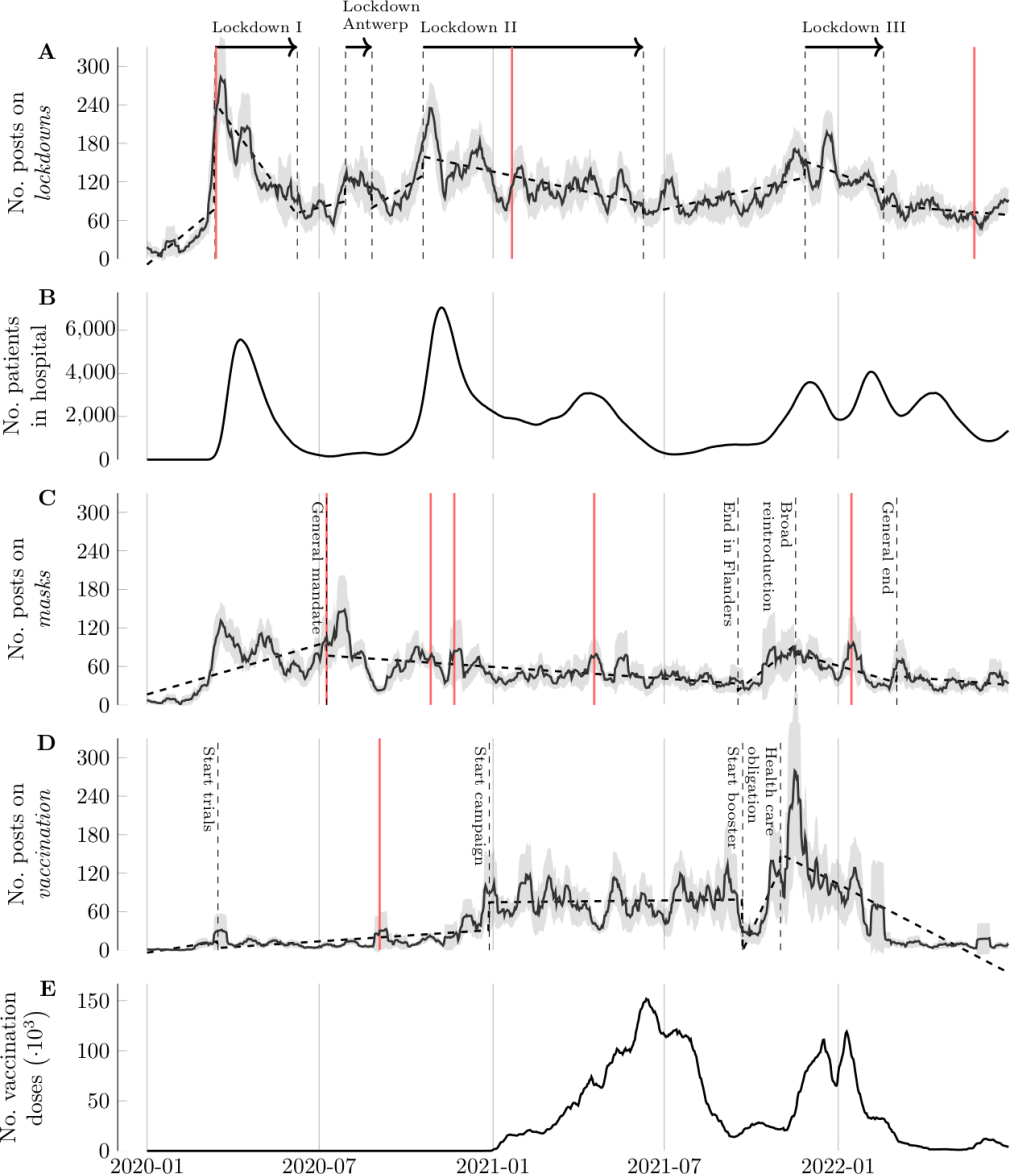
The number of posts per day on r/Belgium on (A) lockdowns, (C) masks, and (D) vaccination; (B) the number of COVID-19 hospitalizations; and (E) vaccination doses in Belgium. All quantities are shown as their 2-week rolling mean. Piecewise linear trends are given by dashed lines. Red vertical lines mark significantly negative days. Important events are denoted by vertical dashed lines or gray regions (Multimedia Appendix 2).

Figure 4 shows posting volume over time for the 3 topics, alongside key events and epidemiological indicators. It gives an overview of posting behavior over the period of the pandemic and associated events. The largest peaks in the number of posts coincided with the external events listed in Table S1 in Multimedia Appendix 2. For example, the start of the first lockdown coincided with the single most active day across all topics, with a rolling mean of 282.79 (SD 126.68) posts on March 19, 2020. Smaller local maxima appeared during the Antwerp lockdown and at the start of the second national one. All events coincided with significant changes in post volume. The piecewise linear trends overlaid on Figure 4 (dashed lines) make these structural changes visible, with the slope and level shifts quantified in Table 2. After these peaks, the volume of posts decreased and stabilized. Three days showed a significantly negative sentiment (days where the median score-weighted sentiment fell below the 0.275 quantile of the topic distribution, with at least 50 comments submitted): at the start of the first lockdown, in the middle of the second national one, and in February 2022, after the end of most mitigation measures. During the first one, negative comments were aimed at “lockdown parties,” social gatherings just before the restrictions came into effect.

**Table 2.** Results of the interrupted time series analysis. Change in level ∆*β*_0*,j*_ and in slope ∆*β*_1*,j*_ at the time of each event *j* per topic, with their 95% CIs.

Events	Dates	∆*β*0*,j (*95% CI)		∆*β*1*,j* (95% CI)	
Lockdowns					
Start lockdown 1	March 13, 2020	*165.17*^a^ (151.39-178.96)		*−3.22* (−3.53 to −2.91)	
End lockdown 1	June 8, 2020	3.82 (−11.32 to 18.96)		*2.36* (1.91-2.81)	
Start lockdown Antwerp	July 29, 2020	*39.85* (19.77 to 59.93)		−1.05 (−2.14 to 0.04)	
End lockdown Antwerp	August 26, 2020	*−30.33* (−50.72 to −9.94)		*1.64* (0.56-2.72)	
Start lockdown II	October 19, 2020	*29.43* (16.24-42.62)		*−1.25* (−1.63 to −0.87)	
End lockdown II	June 9, 2021	*−15.93* (−24.62 to −7.25)		*0.65* (0.57-0.73)	
Start lockdown III	November 27, 2021	*25.16* (13.66-36.67)		*−0.87* (−1.08 to −0.66)	
End lockdown III	February 18, 2022	*−23.90* (−36.02 to −11.77)		*0.42* (0.20-0.64)	
Masks					
General mandate	July 9, 2020	*−20.73* (−27.74 to −13.72)		*−0.53* (−0.58 to −0.47)	
End in Flanders	September 17, 2021	−10.56 (−21.44 to 0.33)		*1.27* (0.97 to 1.56)	
Broad reintroduction	November 17, 2021	*−13.69* (−26.67 to −0.72)		*−1.59* (−1.91 to −1.27)	
General end	March 4, 2022	10.10 (−0.63 to 20.82)		*0.31* (0.15-0.48)	
Vaccination					
First trials	March 16, 2020	−10.36 (−22.61 to 1.90)		−0.14 (−0.39 to 0.11)	
Start campaign	December 28, 2020	*43.29* (35.32-51.26)		*−0.08* (−0.13 to −0.03)	
Start booster	September 22, 2021	*−79.62* (−95.27 to −63.96)		*3.21* (2.57 to 3.86)	
Health care obligation	November 19, 2021	*21.46* (5.20-37.73)		*−4* (−4.64 to −3.35)	

a Italics indicate significant changes.

Discussions on masks began at the onset of the COVID-19 outbreak in Belgium, four months before the announcement of a general mandate (July 9, 2020), which itself did coincide with a small increase in posts, and a significantly negative day. During the latter, negative comments criticized the late decision of the government to introduce the mandate, and people not following it. Activity on mask-related discussions fell after the first lockdown but rose again during the second mandate period (November 2021-March 2022), which included another significantly negative day. Another 3 significantly negative days occurred during the first mandate. All events were associated with a significant change in trend, and after the 2 moments the mandate was expanded, the level changed significantly as well (Table 2). Post volume generally increased before mask mandates were tightened and started decreasing afterward.

Similar to the lockdown-related events, major vaccine-related events or news corresponded well with the volume of vaccination posts on Reddit. Broad discussions did not start until the run-up to the vaccination campaign, with the only nonsignificant change in level or volume occurring at the start of the trials (Table 2). The announcement that vaccination would be mandatory for health care personnel preceded the month with the highest posting activity, culminating in a rolling mean peak of 279.64 (SD 124.17) posts on November 16, 2021. The single significantly negative day, September 3, 2020, fell during the trials. Comments reacted to an article stating that 30% of the Belgian population would refuse a COVID-19 vaccine [60]. The negative reactions targeted both refusers and the “rushed” development of the new mRNA technology.

### Topic Contagion

**Figure 5.**
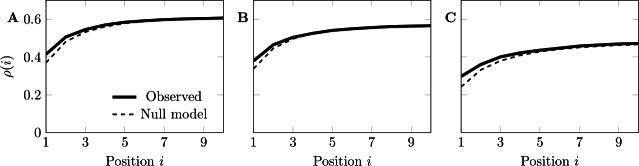
Proportion of users ρ(i) that have at least one initiated post in the first i discussions for the three COVID-19 mitigation topics—(A) lockdowns (9987 users), (B) masks (6824 users), and (C) vaccination (5552 users)—in r/Belgium (January 2020-June 2022). The full line represents the observed proportion, and the dashed line represents the expected proportion under the null model.

We tested whether prior participation in a discussion on a topic increases the probability of subsequently initiating a new one, which serves as an indication of social contagion. Under the null hypothesis (no contagion), initiations and participations are ordered uniformly at random. Under contagion, on the other hand, participation would increase the probability of later initiating a new discussion, causing participations to accumulate earlier in users’ sequences. Figure 5 shows the proportion of users with at least one initiation among their first discussions, relative to those with a participation or initiation, ρ(i). The solid line represents the observed cumulative proportion and the dashed line is the expected proportion under the null model. If contagion were present, *ρ*(*i*) would lie below the null for small *i*, reflecting an accumulation of participations before the first initiation. For all topics, the observed curve lies above the null at low values of i, indicating initiations occurred earlier in posting sequences than expected. This contradicts the contagion hypothesis: initiations were more likely near the start of sequences. Consequently, users who participated in a discussion were not more likely to subsequently initiate one on the same topic. No evidence of social contagion was found for discussion initiation on the 3 mitigation topics within r/Belgium, neither simple nor complex. As ρ(i) converges, it approaches the proportion of users with at least one initiation: 0.612 for lockdowns, 0.572 for masks, and 0.481 for vaccination. Hence, not only did discussions on vaccination appear later than those on the other topics, they were initiated by a relatively smaller fraction of the user base.

### Sentiment Homophily

**Figure 6.**
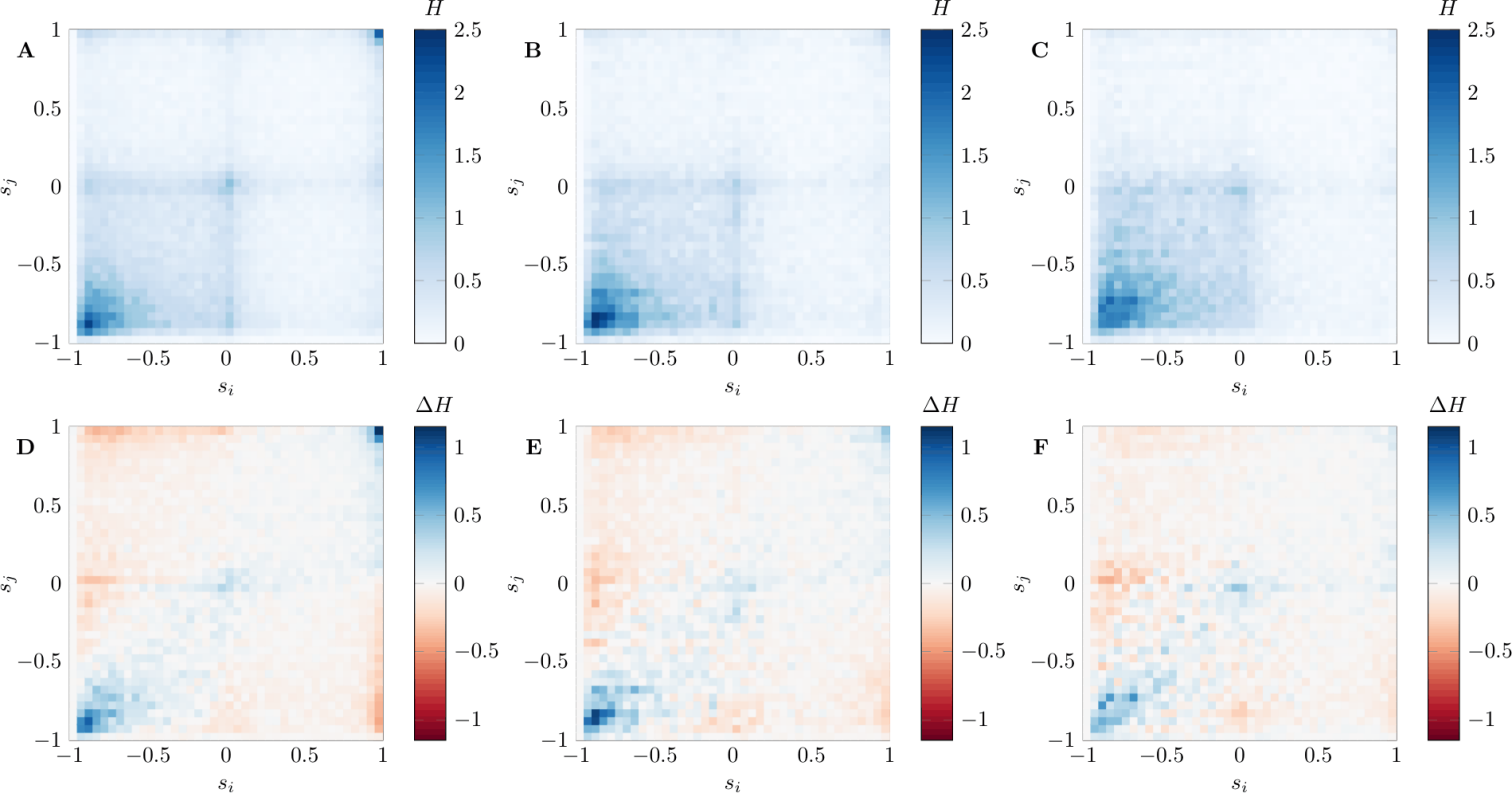
Normalized histograms of (A, B, C) observed sentiment interactions on r/Belgium (January 2020-June 2022) and (D, E, F) the difference with the null model for the COVID-19 mitigation topics (A, D) lockdowns (94,494 pairs), (B, E) masks (48,500 pairs), and (C, F) vaccination (41,700 pairs).

We examined the relationship between the sentiment of a comment and that of its parent, looking for correlations between these, namely homophily. Figure 6 presents heatmaps of comment-parent sentiment pairs *H* and ∆*H* for each topic. Across all topics, the center of mass of observed interactions H lay in the fourth quadrant, with a global maximum near (−1,−1), indicating most posts expressed negative sentiment. For lockdowns, another local maximum appears in the first quadrant corner. Discussions around masks and vaccination exhibited consensus (predominantly negative), while those on lockdowns showed polarization (negative replies to negative, positive to positive) [29].

Both consensus and polarization fall under the broader term homophily, the tendency to interact with others expressing similar sentiment. Homophily is expected when the underlying distribution is narrow, offering few interaction alternatives. However, the heatmaps of ΔH in Figure 6 demonstrate homophily for all topics, irrespective of sentiment distribution shape. Positive values center around the diagonal, indicating more interactions between similar sentiment than expected. Negative values appear in the second and third quadrants, and in regions representing interactions between negative and neutral sentiment. Discussions on lockdowns were the most polarized, with clear peaks in the equally-signed corners. The discrepancy between observed and expected values reveals greater polarization than H alone suggested for masks, with positive values of ΔH near (1,1). The homophily measure h (Equation A.7 in Multimedia Appendix 3) is 0.246, 0.212, and 0.144 for lockdowns, masks, and vaccination, respectively. As Figure 6 suggests, homophily is most pronounced for lockdowns and least pronounced for vaccination. This mirrors the pattern reported in sections *Temporal Analysis* and *Topic Contagion*, where lockdowns and vaccination produce the most differing results, with masks showing more similarities to lockdowns than to vaccination.

**Figure 7.**
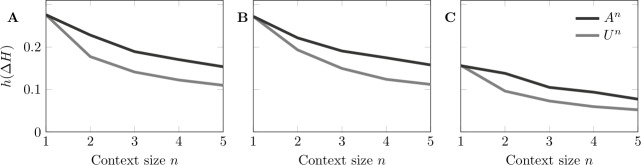
Measure of homophily *h* between the sentiment of a comment and its context size *n* (ancestral context: black; user context: gray) for the COVID-19 mitigation topics (A) lockdowns (9067 comments), (B) masks (5372), and (C) vaccination (4893 comments) on r/Belgium (January 2020-June 2022).

We examined which context type exhibited stronger homophily: ancestral or $A_{k}^{n}$ user $U_{k}^{n}$. The former comprises posts the focal user most recently read, while the latter comprises posts with which the user most recently interacted. For this, a subsample of comments allowing a sufficient size for both contexts was considered (9067, 5372, and 4893 comments for lockdowns, masks, and vaccination, respectively). Figure 7 presents the homophily h measure for all 3 topics and both context types as a function of context size n, the number of preceding posts that are taken into account. With h being 0.276 (lockdowns), 0.272 (masks), and 0.156 (vaccination) for n=1, homophily in this subset is more pronounced, but the relative ordering of topics persists. The homophilic effect decreased with increasing context size n, regardless of type. Homophily within the ancestral context consistently exceeded that in the user context for all n. Comment sentiment was thus more strongly related to the immediate posting environment than to sentiment that previously prompted replies from the focal user. Comments on vaccination exhibited less homophily than the other topics.

### Sentiment Evolution Model

The SLEBC model (Equation 1) was set up to give mechanistic insights into the results of section *Sentiment Homophily*. To fit the parameters of this model and its alternatives, we considered 209, 101, and 98 users for lockdowns, masks, and vaccination, respectively, each with at least 40 comments. These users were the most active and thus yielded sufficient data, though the homophily within this population differed from the complete set (relative difference of −10.5% for lockdowns, +12.5% for masks, and +13.7% for vaccination). Consequently, the results might not apply to the less active users.

Table 3 compares the performance of the 3 models, with lower WAIC and |Δh| indicating better model fit. All models systematically underestimated homophily, as indicated by negative Δh, with only SLEBC having 95% credible intervals that encompass zero. The SLEBC model outperformed both alternatives across all topics, achieving the lowest WAIC values and homophily deficits closest to zero. The two alternatives each have their weaknesses. The linear model (Equation A.9) in Multimedia Appendix 3) achieved comparable predictive power to SLEBC, but substantially underestimated homophily. The stateless model (Equation A.10 in Multimedia Appendix 3), which ablated the latent state, achieved a worse fit, but more accurately predicted homophily. These results suggest the bounded confidence kernel is better suited to model the sentiment homophily, with the model lacking it, underestimating *h* more severely. Moreover, the latent state improves capturing the sentiment distribution over time by accounting for fluctuating expressed sentiment.

**Table 3.** Model comparison showing Watanabe-Akaike information criterion and homophily difference ∆*h* (with 95% credible interval [CrI]) for the smooth latent-expressed bounded confidence model and its two alternatives, the linear model *L_α_* and the stateless model $\bar{e}$. Applied to highly active users (≥40 comments) of r/Belgium (January 2020-June 2022) in COVID-19 mitigation topics: lockdowns (209 users*,* 16,762 comments), masks (101 users, 4277 comments), and vaccination (98 users, 3169 comments).

Model	WAIC^a^	∆*h* (95% CrI)	
Lockdowns			
*L_α_* (Equation A.9)	−21.11	−0.0973 (−0.1084 to −0.0720)	
$\bar{e}$ (Equation A.10)	692	−0.0702 (−0.1078 to −0.0441)	
SLEBC (Equation 1)	−28.5	−0.0273 (−0.0511 to 0.0015)	
Masks			
*L_α_* (Equation A.9)	−17.4	−0.1317 (−0.1651 to −0.0972)	
$\bar{e}$ (Equation A.10)	169	−0.0759 (−0.1534 to −0.0276)	
SLEBC^b^ (Equation 1)	−18.4	−0.0671 (−0.0816 to 0.0103)	
Vaccination			
*L_α_* (Equation A.9)	−20.6	−0.1032 (−0.1393 to −0.0732)	
$\bar{e}$ (Equation A.10)	6.81	−0.0672 (−0.1395 to −0.0410)	
SLEBC (Equation 1)	−21.2	−0.0337 (−0.0829 to 0.0046)	

a WAIC: Watanabe-Akaike information criterion.

b SLEBC: smooth latent-expressed bounded confidence.

**Figure 8.**
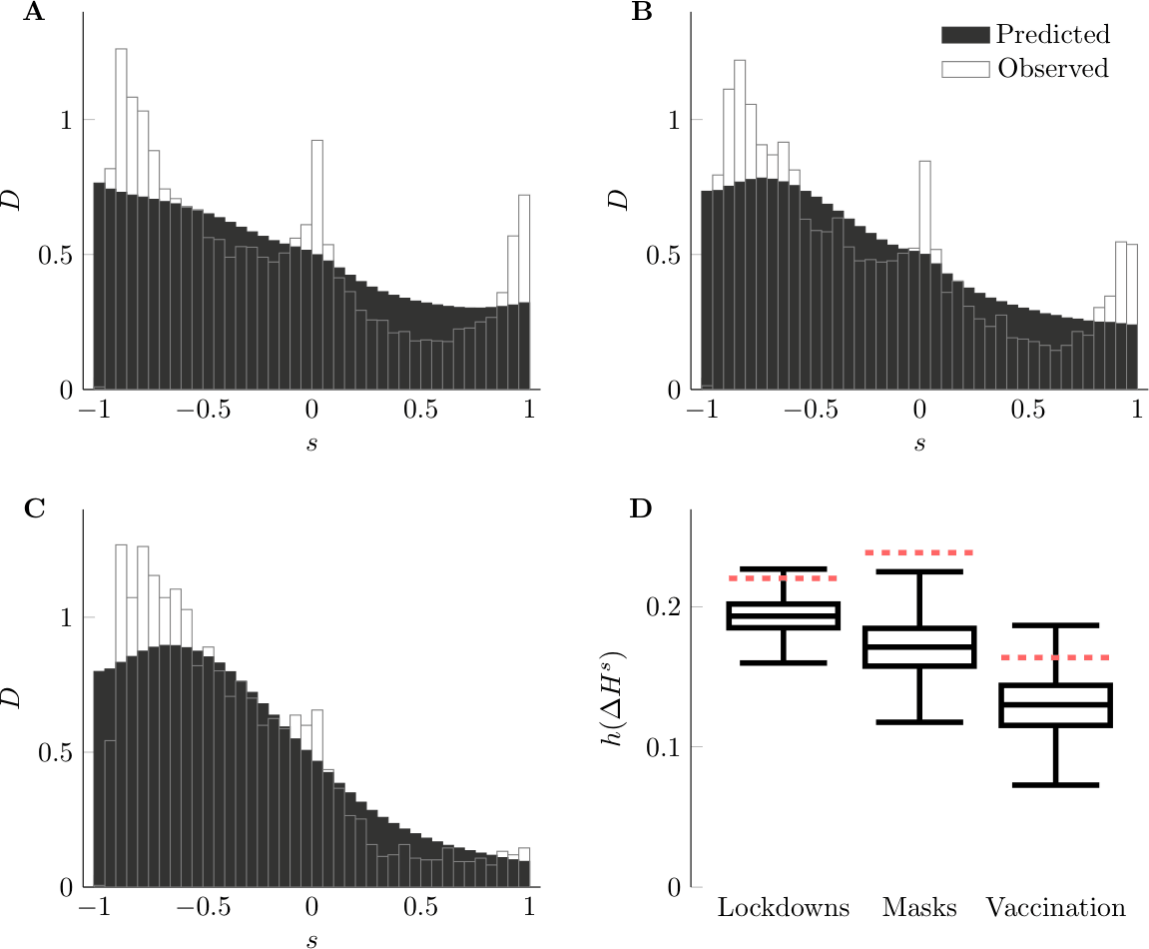
Outcomes of the fitted posterior of the smooth latent-expressed bounded confidence model on highly active users (≥40 comments) of r/Belgium (January 2020-June 2022). Normalized distributions *D* of the predicted sentiment for the topics (A) lockdowns (209 users, 16,762 comments), (B) masks (101 users, 4277 comments), and (C) vaccination (98 users, 3169 comments) in the smooth latent-expressed bounded confidence model, compared to the observed sentiment. (D) The predicted values of *h* by the smooth latent-expressed bounded confidence model, with minimums, maximums, and 0.25, 0.5, and 0.75 quantiles. The dashed red line represents the observed value.

Figure 8 compares per topic the posterior sentiment distributions and homophily values obtained from the SLEBC model to those observed in the data. The SLEBC model predicted sentiment distributions that placed most mass near the negative extreme, while spreading more evenly over their support than observed distributions and lacking their tri-modal structure (Figure 8A-C). In particular, the local maximum around zero (neutral sentiment) present in the data was not captured. On the positive extreme, both observed and predicted distributions exhibited a local maximum for lockdowns, though this peak was substantially more pronounced in the data. Applying the homophily analysis from section *Sentiment Homophily* to the predictions showed that the SLEBC model generated homophily (Figure 8-D), but underestimated it for all topics, with median values exceeding 0.75 quantiles of predicted values for all topics, and even the maximum for masks.

Table 4 presents a quantitative summary of the sentiment threshold ϵ and update strength parameters $\alpha_{u}$ and in the SLEBC model (Equation 1). For all topics and most users, expressed sentiment was more strongly influenced by parent comment sentiment than latent sentiment was by other interactions, as κ exceeded one half, although only marginally for vaccination. Therefore, adaptation to parent comment sentiment constituted an important mechanism behind the observed homophily, consistent with findings in section *Sentiment Homophily*. The latent update strength $\alpha_{u}$ varied less across topics and users within a topic, as shown by its narrower quantile intervals, indicating that the degree of expressed sentiment adaptation to the parent depended more on user-specific behavior.

**Table 4.** The population mean values of $\epsilon ,\alpha_{u}$, and $a_{e}$ with their 95% quantile intervals (95% QIs) in the smooth latent-expressed bounded confidence model. Equation 1 sampled from the posterior distribution, together with κ, the proportion of users for which $P(\alpha_{u,i}§lt;\alpha_{e,i})§gt;P(\alpha_{u,i}§gt;\alpha_{e,i})$, with *P*<.05 for highly active users, in COVID-19 mitigation discussions on r/Belgium (January 2020-June 2022) on lockdowns (209 users)*,* masks (101 users), and vaccination (98 users).

Topic	ϵ	95% QI	αᵤ	95% QI	αₑ	95% QI	*κ*
Lockdowns	0.973	(0.367-1.566)	0.414	(0.126-0.706)	0.871	(0.269-3.694)	0.742
Masks	0.874	(0.372-1.510)	0.417	(0.238-0.609)	0.656	(0.265-1.933)	0.703
Vaccination	0.971	(0.445-1.484)	0.425	(0.139-0.788)	0.508	(0.234-1.389)	0.510

The mean sentiment threshold ϵ is lower for masks than the other two topics, while the quantile interval for vaccination is the narrowest. For all topics, the mean value is just under half of the maximum meaningful value, two. The quantile intervals indicate that only a small number of users have very low or very high ones. The SLEBC model thus infers that users were moderately open to influence: they adapted their sentiment toward that of others unless opinions diverged by more than roughly half the scale, for example, between negative and neutral. In contrast, only a limited number of users displayed no sentiment adaptation (ϵ≈0), or indiscriminate adaptation (ϵ≈2).

**Figure 9.**
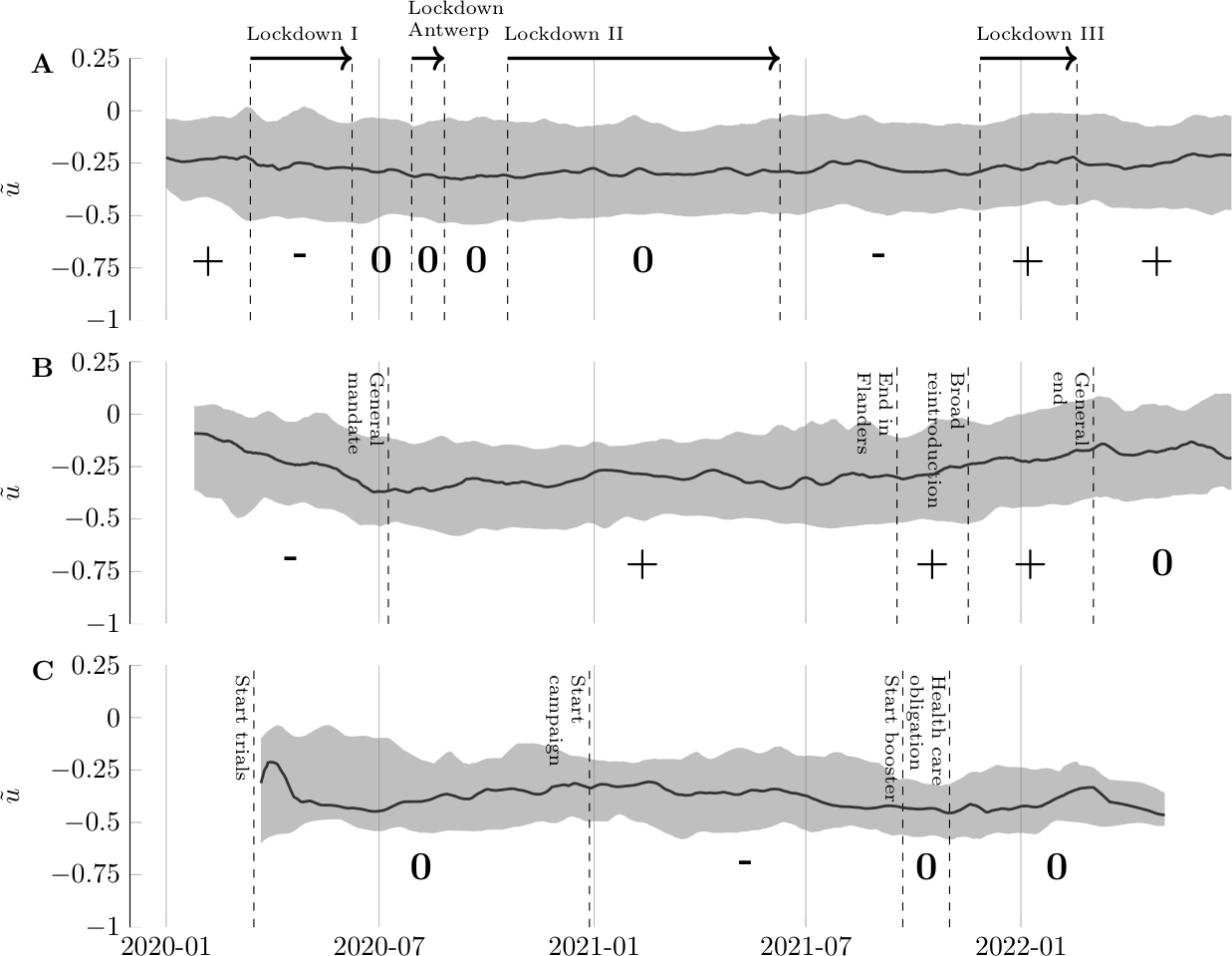
Median latent sentiment u ~ of highly active users over time (full black curve) for the COVID-19 mitigation topics (A) lockdowns (209 users), (B) masks (101 users), and (C) vaccination (98 users) on r/Belgium (January 2020-June 2022). The area between the first and third quartile is shaded. Hamed-Rao monotonic test results are given per period delimited by events in Multimedia Appendix 2 (+: increasing, -: decreasing, 0: no significant trend).

A key feature of the SLEBC model is its ability to separate the influence of a user’s latent sentiment from that of the parent comment on the expressed sentiment. For assessing sentiment toward a mitigation measure, the former is particularly informative, as it captures the less noisy, long-term trends. Figure 9 presents the evolution of the inferred latent state of the SLEBC model, *u,* for the studied population. For masks, median latent sentiment declined during the first half of 2020 before gradually increasing. For lockdowns, latent sentiment remained stably negative throughout the study period. The Hamed-Rao test identified both increasing and decreasing trends at the start and end of the considered period; however, the absolute change was small. This is the case for vaccination as well, although sentiment initially showed a short positive tendency around the start of the trials.

## Discussion

### Principal Findings

This study analyzed discussions on COVID-19 mitigation measures on the Belgian Reddit community (r/Belgium) from January 2020 to June 2022, examining how discussion volume and sentiment evolved around lockdowns, masks, and vaccination. We found that discussion volume was primarily associated with external events such as policy announcements and media coverage rather than with social contagion within the platform. Peaks in posting activity aligned with major policy changes, including the first lockdown and the announcement of mandatory vaccination for health care personnel, suggesting that traditional media and official communications may play an important role in shaping health discourse in r/Belgium, as has been confirmed on Twitter [11,61] and other Reddit communities [62]. This contrasts with observations for other online behaviors where complex social contagion mechanisms have been documented [34].

Although topic-level contagion was not significant, sentiment exhibited homophily, with users adapting their sentiment to match that of their parent comment. The observed homophily measure *h* ranged from 0.144 to 0.246 across the 3 topics, confirming patterns previously documented on Reddit [16,43]. Our analysis suggested that this homophily operated primarily through immediate conversational adaptation rather than through selective interaction with like-minded users. To capture this dynamic, we developed the SLEBC model, which distinguishes between a user’s unobserved latent sentiment and the observed sentiment expressed in comments. The model successfully reproduced observed patterns and revealed that most users (51%‐74% depending on topic) adjusted their expressed sentiment more strongly to match that of the parent than they updated their latent sentiment state.

The SLEBC model, building on bounded confidence modeling [48], outperformed linear models, indicating that sentiment alignment occurred only when users encountered other users with sufficiently similar sentiment. Our results expand on the existing literature on using the bounded confidence model in steady-state opinion distributions on social media [29], and spatial patterns of vaccine hesitancy [36]. Nevertheless, a linear model explicitly using social network structure has been successfully calibrated on Italian vaccine hesitancy survey data, with the authors naming the bounded confidence model as a possible extension [63]. By also incorporating a latent sentiment state, the SLEBC model corrected for the fluctuating nature of the expressed sentiment and better inferred the observed distribution of sentiment over time. This latent state may better represent a user’s underlying sentiment trajectory than their fluctuating expressions alone, making it a stronger signal for relating online discourse to behavioral indicators. Discussions on different mitigation measures showed distinct patterns on r/Belgium, similarly to Twitter [20]. Discussions on lockdowns dominated in volume and emerged when restrictions were first imposed. This aligns with the increase in the volume of COVID-19-related posts observed in other country-related subreddits during a lockdown period [28]. Masks were discussed from the pandemic’s onset, before any official mandate. Vaccination, by contrast, did not attract broad discussion until the run-up to the campaign. A possible explanation for the temporal differences is that mask wearing was a personal choice before the official mandate, whereas vaccination was impossible before the government launched the campaign.

The 3 topics also differed in sentiment. Discussions on lockdowns showed stronger polarization with distinct positive and negative sentiment clusters. In contrast, discussions on masks and vaccination both exhibited predominantly negative sentiment, though vaccination showed weaker homophily (*h*=0.144) than masks (*h*=0.212). This is mirrored in the inferred parameters of the SLEBC model. For lockdowns, the mean external update strength is strongest, followed by masks and vaccination. In contrast, population distributions of sentiment thresholds and latent update strengths showed more similarities over the topics. Aforementioned differences underscore the importance of analyzing mitigation topics separately rather than aggregating them into a single “COVID-19 sentiment” variable.

Existing coupled epidemiological-social models may implicitly treat social media activity as a direct proxy for the infection dynamics, whereas our results indicate that a communication layer should be considered as well [64]. In particular, modeling of discussion volume may benefit from an intermediate layer representing official communications and traditional media such as the OxCGRT stringency index [65] or GDELT dataset [66]. Users can then react to activity in this layer, allowing for an increase in discussion volume without within-platform contagion. Sentiment dynamics are better represented by complex contagion models such as bounded confidence kernels rather than by simple ones. The methodological framework of combining topic modeling, sentiment analysis, null model comparisons, and mechanistic modeling provides a template for digital health surveillance deployment during health emergencies, with established platforms like Reddit offering advantages as data infrastructure and analysis pipelines can be prepared in advance.

### Limitations and Future Work

This study has several limitations. The r/Belgium community represents a self-selected, nonrepresentative sample of the Belgian population. Reddit users tend to belong to a specific demographic, often young and male [31]. This effect intersects with our focus on English-language posts (approximately 75% of the dataset). However, because English acts as a lingua franca on r/Belgium (and the majority of other Reddit communities) that bridges the country’s distinct linguistic communities, restricting the analysis to English avoids introducing confounding variables from isolated subgroups that communicate exclusively in Dutch, French, or German. Moreover, SLEBC parameters were inferred from highly active users, whose homophily values differed from the full sample. Therefore, inferred parameters and latent trajectories may not represent the sentiment states of casual users. Nevertheless, qualitative findings such as the adaptation of expressed sentiment to the parent post are furthermore supported by the full dataset analysis in section *Sentiment Homophily*. Still, our findings represent platform- and user-specific dynamics that may not generalize, as highly active, English-proficient Reddit users may differ systematically in demographics, political orientation, or health attitudes from other communities. Applying our approach across different communities and platforms would help clarify how specific platform architectures influence sentiment homophily [15,25,61].

Sentiment analysis captures linguistic tone rather than explicit policy support, introducing ambiguity where negative sentiment could reflect criticism of either insufficient or excessive measures. The rapid increase in ability and availability of large language models can enhance our methodology by extracting more nuanced signals such as support for public health measures and willingness to adhere. The inferred latent states are unobservable mathematical constructs. While our model successfully reconstructs latent sentiment, it remains to be established whether these trajectories serve as reliable proxies for true offline attitudes. Future work could address this by relating the inferred latent states to behavioral surveys or adherence metrics.

### Conclusions

Our study showed that discussions on COVID-19 mitigation measures on Reddit’s r/Belgium were not associated with social contagion within the platform but aligned with external events and traditional media. In contrast, sentiment dynamics were shaped by within-thread interactions. Users exhibited sentiment homophily by adapting their expressed sentiment to match that of preceding comments in the thread. The SLEBC model captures this by distinguishing between an unobserved latent sentiment state and sentiment expressed in comments, suggesting that expressed sentiment adapts strongly to align with that of the parent comment and therefore may poorly reflect users’ underlying sentiment.

These findings have implications for Reddit-based digital health surveillance and epidemic modeling that uses social media signals. First, rather than assuming purely endogenous social contagion, models benefit from an intermediate communication layer of external official communications and traditional media as the driver of topic emergence. Second, when integrating social sentiment as a proxy for public adherence, models should account for interaction structure. In COVID-19 mitigation discussions on r/Belgium, expressed sentiment adapted primarily to the immediate parent comment rather than to a user’s broader interaction history, and this adaptation was better captured by a bounded confidence kernel than by a linear update, with the latter substantially underestimating observed homophily. Therefore, raw expressed sentiment is a noisy, perturbed signal. The inferred latent trajectory is more stable and, if validated against behavioral outcomes, is a stronger candidate for model input. Finally, mitigation measures exhibited distinct patterns in our research and should not be collapsed into a single “COVID-19 sentiment” variable, which would obscure relevant differences.

## Supplementary material

10.2196/87723 Multimedia Appendix 1 Glossary.

10.2196/87723 Multimedia Appendix 2 Overview of selected events included in this study.

10.2196/87723 Multimedia Appendix 3 Supporting equations and methods.

10.2196/87723 Multimedia Appendix 4 Sensitivity analysis of homophily measure.

## Abbreviations

BERT: bidirectional encoder representations from transformers
RoBERTa: robustly optimized BERT pretraining approach
SLEBC: smooth latent-expressed bounded confidence
WAIC: Watanabe-Akaike information criterion
