# Supplementary material for: Social Contagion in COVID-19 Discussions Within the Belgian Reddit Community: Statistical and Modeling Study
Source: J Med Internet Res. 2026 Jul 29;28:e87723. doi: 10.2196/87723 (PMC13419282; doi:10.2196/87723)
Supplement: Multimedia Appendix 1 [file jmir-v28-e87723-s001.pdf]

Glossary

Term	Symbol	Description
Reddit		Social media platform where users can post links, images, and text.
Subreddit		Community within Reddit, denoted by prefix r/.
Submission		Top-level post on Reddit.
Comment		Reply to a submission or other comment.
Post		Submission or comment.
Thread		A submission with all its comments.
Parent		Submission or comment a comment is a reply to.
Ancestors		All successive parents of a comment up to and including the submission.
Discussion		Part of a thread with a common topic.
Initiator		Creator of a post that does not share its topic with any of its ancestors.
Participant		User in a discussion that is not the initiator.
Ancestral context	A_k^n	The n closest ancestors of a comment k.
User context	U_k^n	The parents of comment k and of the $n-1$ preceding comments made by same author on the same topic.
mbert-ctbt		mbert-corona-tweets-belgium-topics, the used topic model [20].
roberta-tbsl		twitter-roberta-base-sentiment-latest, the used sentiment model [42].
Sentiment	s	The emotional tone of a text classified by roberta-tbsl.
Homophily	h	Tendency of individuals to associate with similar ones, here specifically with similar sentiment.
Consensus		A pattern of homophily where users predominantly express similar sentiment, leading to a unimodal distribution
Polarization		A pattern of homophily where two clusters of users express different sentiment, leading to a bimodal distribution
SLEBC		Smooth Latent-Expressed Bounded Confidence.
