# Supplementary material for: Social Contagion in COVID-19 Discussions Within the Belgian Reddit Community: Statistical and Modeling Study
Source: J Med Internet Res. 2026 Jul 29;28:e87723. doi: 10.2196/87723 (PMC13419282; doi:10.2196/87723)
Supplement: Multimedia Appendix 2 [file jmir-v28-e87723-s002.pdf]

Main Events

Event	Date	Description	Ref.
Lockdowns			
Lockdown I	2020-03-13	Closure of restaurants, pubs, schools, entertainment venues and non-essential stores, part of a European wave of similar national lockdowns.	[1, 2]
	2020-06-08	Reopening of culinary, cultural, amusement and religious establishments.	[3]
Lockdown Antwerp	2020-07-29	Introduction of a curfew and restriction of four persons per table in bars and restaurants in the Antwerp province.	[4]
	2020-08-26	End of specific restrictions in the province of Antwerp.	[5]
Lockdown II	2020-10-19	Closure of restaurants and pubs, reduction of close contacts to one and introduction of a curfew.	[6]
	2021-06-09	Reopening of restaurants, pubs and cultural and entertainment venues.	[7]
Lockdown III	2021-11-27	Restriction of the number of persons per table and opening hours in restaurant and bars. Prohibition of private and events without seating.	[8]
	2022-02-18	Restart of public events and unrestricted opening hours for bars and restaurants.	[9]
Masks			
General mandate	2020-07-09	Announcement of a mask mandate for commercial, cultural and religious venues, following Spain and France	[10, 2]
End in Flanders	2021-09-17	Announcement of reduction of federal mandate to public transport, big events, close contact professions and healthcare. In practice only happened in the region of Flanders.	[11]
Broad reintroduction	2021-11-17	Announcement of extension of the federal mask mandate to public and commercial venues.	[12]
General end	2022-03-04	Announcement of mask obligation limited to public transport and healthcare from 7th of March.	[13]
Vaccination			
First trials	2020-03-16	Clinical testing on human starts.	[14]
Start campaign	2020-12-28	First vaccination in Belgium.	[15]
Start booster	2021-09-22	Decision to allow third vaccinations for retirement home inhabitants.	[16]
Healthcare obligation	2021-11-19	Federal government reaches agreement about obligation of vaccination in healthcare.	[17]

Table S1: Description of the events shown in Figure 4 [45]. Except where explicitly stated, all measures were imposed by the federal government and hence applicable to the whole country.

References

1. Binnenlandse Zaken, Ministerieel besluit houdende dringende maatregelen om de verspreiding van het coronavirus COVID-19 te beperken, eJustice, https://www.ejustice.just.fgov.be/eli/besluit/2020/03/13/2020030303/justel (Mar. 2020), accessed on 2024-11-15.
2. T. Hale, N. Angrist, R. Goldszmidt, B. Kira, A. Petherick, T. Phillips, S. Webster, E. Cameron-Blake, L. Hallas, S. Majumdar, H. Tatlow, A global panel database of pandemic policies (Oxford COVID-19 Government Response Tracker), Nature Human Behaviour 5 (4) (2021) 529–538. doi:10.1038/s41562-021-01079-8.
3. S. Wilmès, Start van fase 3 van het afbouwplan vanaf 8 juni, https://www.info-coronavirus.be/nl/news/nvr-0306/ (Jun. 2020) , accessed on 2024-11-15.
4. T. Santens, Boete tot 1.600 euro voor wie avondklok schendt, sporten mag zonder masker: dit is nu vastgelegd in Antwerpen, VRT Nieuws, https://www.vrt.be/vrtnws/nl/2020/07/29/antwerpse-maatregelen/ (Jul. 2020), accessed on 2024-11-15.
5. R. Arnoudt, Antwerpse maatregelen bijgestuurd: Horeca langer open, avondklok wordt nachtklok, evenementen weer mogelijk, VRT Nieuws, https://www.vrt.be/vrtnws/nl/2020/08/12/antwerpse-gouverneur-maakt-een-nachtklok-van-de-avondklok/ (Aug. 2020), accessed on 2024-11-15.
6. A. De Croo, COVID-19 alarmniveau gaat in: strengere regels vanaf maandag 19 oktober, https://www.premier.be/nl/covid-19-alarmniveau-gaat-strengere-regels-vanaf-maandag-19-oktober (Oct. 2020), accessed on 2024-11-15.
7. Overlegcomité, Zomerplan: in vier stappen naar normaler levens, https://www.belgium.be/nl/nieuws/2021/zomerplan___vier_stappen_naar_normaler_levens (May 2021), accessed on 2024-11-15.
8. Binnenlandse Zaken, Koninklijk besluit houdende wijziging van het koninklijk besluit van 28 oktober 2021 houdende de nodige maatregelen van bestuurlijke politie teneinde de gevolgen voor de volksgezondheid van de afgekondigde epidemische noodsituatie betreffende de coronavirus COVID-19 pandemie te voorkomen of te beperken, eJustice, https://www.ejustice.just.fgov.be/eli/besluit/2021/11/27/2021043241/justel (Nov. 2021), accessed on 2024-11-15.
9. Overlegcomité, Code oranje vanaf 18 februari 2022: geen sluitingsuur horeca meer, nachtleven open, https://www.belgium.be/nl/nieuws/2022/code_oranje_vanaf_18_februari_2022_geen_sluitingsuur_horeca_meer_nachtleven_open (Feb. 2022), accessed on 2024-11-15.
10. TT, Mondmaskers vanaf zaterdag verplicht in alle winkels, bioscopen, gebedshuizen, bibliotheken en musea, HLN, https:// www.hln.be/binnenland/mondmaskers-vanaf-zaterdag-verplicht-in-alle-winkels-bioscopen-gebedshuizen-bibliotheken-en-musea (Jul. 2020), accessed on 2024-11-15.
11. Overlegcomité, Overlegcomité bepaalt federale sokkel mondmaskerplicht, https://www.belgium.be/nl/nieuws/2021/ overlegcomite_bepaalt_federale_sokkel_mondmaskerplicht (Sep. 2021), accessed on 2024-11-15.
12. Overlegcomité, Overlegcomité: voortaan brede mondmaskerplicht en verplicht telewerk, https://www.belgium.be/nl/ nieuws/2021/overlegcomite_voortaan_brede_mondmaskerplicht_en_verplicht_telewerk (Nov. 2021), accessed on 2024-11-15.
13. Binnenlandse Zaken, Koninklijk besluit houdende wijziging van het koninklijk besluit van 28 oktober 2021 houdende de nodige maatregelen van bestuurlijke politie teneinde de gevolgen voor de volksgezondheid van de afgekondigde epidemische noodsituatie betreffende de coronavirus covid-19 pandemie te voorkomen of te beperken, eJustice, https://www.ejustice.just.fgov.be/eli/besluit/2022/03/05/2022040532/justel (Mar. 2022) , accessed on 2024-11-15.

14. T. Thanh Le, Z. Andreadakis, A. Kumar, R. Gómez Román, S. Tollefsen, M. Saville, S. Mayhew, The COVID-19 vaccine development landscape, Nature Reviews Drug Discovery 19 (5) (2020) 305–306. doi:10.1038/d41573-020-00073-5.
15. E. Maerevoet, Jos Hermans (96) krijgt als eerste Vlaming vaccin: "Blij dat ik spuit krijg, ik wil 100 worden", VRT Nieuws, https://www.vrt.be/vrtnws/nl/2020/12/22/jos-hermans-96-ik-ben-blij-dat-ik-de-spuit-ga-krijgen-wil-1/ (Dec. 2020), accessed on 2024-11-15.
16. R. Arnoudt, Derde prik voor bewoners woonzorgcentra, nog geen beslissing over thuiswonende 85-plussers, VRT Nieuws, https://www.vrt.be/vrtnws/nl/2021/09/22/bewoners-van-woonzorgcentra-krijgen-derde-prik-coronavaccin/ (Sep. 2021), accessed on 2024-11-15.
17. G. Paelinck, Federale regering bereikt compromis over verplichte vaccinatie in de zorg, VRT Nieuws, https://www.vrt.be/vrtnws/nl/2021/11/19/akkoord-vaccin/ (Nov. 2021), accessed on 2024-11-15.
