# Supplementary material for: Social Contagion in COVID-19 Discussions Within the Belgian Reddit Community: Statistical and Modeling Study
Source: J Med Internet Res. 2026 Jul 29;28:e87723. doi: 10.2196/87723 (PMC13419282; doi:10.2196/87723)
Supplement: Multimedia Appendix 3 [file jmir-v28-e87723-s003.pdf]

Supporting Equations and Methods

Interrupted Time Series Analysis (Section 2.2)

The linear interrupted time series analysis uses an augmented design matrix to find changes in level (β_0) and slope (β_1) after each designated dates. Let t denote the time (in days) since the start of the time series, and let $\{d_1, d_2, \ldots, d_m\}$ be the set of event dates. The series to fit is then

$$Y(t) = \beta_0 + \beta_1 t + \sum_{j=1}^{m} D_j(t) \cdot \left(\Delta\beta_{0,j} + \Delta\beta_{1,j} t\right), \qquad (A.1)$$

where D_j is the indicator function with $D_j(t) = 1$ if $t \geq d$ and 0 otherwise. Ordinary least squares regression of daily post counts Y yields coefficients β and $\Delta\beta$. For each event j, the level change $\Delta\beta_{0,j}$ and the slope change $\Delta\beta_{1,j}$ give the change in linear trend. Confidence intervals are computed from the covariance matrix.

Weighted Sentiment (Section 2.2)

For each topic x, the weighted sentiment set is defined as

$$S^x = \{v_k s_k \mid \text{post } k \text{ is about topic } x\}, \qquad (A.2)$$

where s_k is the sentiment and v_k is the Reddit score of post k and per day as

$$S_d^x = \{v_k s_k \in S^x \mid \text{post } k \text{ is made on date } d\}. \qquad (A.3)$$

A day d was marked as significantly negative if the median of S_d^x fell below the 0.275 quantile of S^x and at least 50 comments were submitted that day.

Topic Contagion Null Model (Section 2.3)

Given n_I initiations and n_P participations in a user's posting sequence, the total number of orderings under the null model (uniform over all sequences) is

$$N(n_I, n_P) = \binom{n_I + n_P}{n_I}. \qquad (A.4)$$

The number of sequences in which the i-th post is the first initiation (requiring the first $i - 1$ posts to be participations, the i-th to be an initiation, and the rest in any order) is

$$T(i \mid n_I, n_P) = \binom{n_I + n_P - i}{n_I - i}. \qquad (A.5)$$

The null probability is therefore

$$P(i \mid n_I, n_P) = \frac{T(i \mid n_I, n_P)}{N(n_I, n_P)}. \qquad (A.6)$$

The homophily measure (Section 2.4)

The exact formulation of homophily measure h in function of ΔH, the difference between the 2D histograms of observed sentiment-interactions and those expected under our null model, reads

$$h(\Delta H) = w_H^2 \sum_{l,m} \Delta H_{l,m}(1 - 2|l - m|), \tag{A.7}$$

with a summation over all bin midpoints l, m in ΔH, and $w_H{}^2 = 0.0025$ being the bin area for which a sensitivity analysis is given in Appendix B.

SLEBC and Alternative Models (Section 2.5)

The smooth bounded confidence kernel used in the SLEBC model is given by

$$B_{\alpha,\epsilon}(s_1, s_2) = s_1 + \frac{\alpha(s_2 - s_1)}{1 + \exp\left(\eta((s_2 - s_1)^2 - \epsilon^2)\right)}, \tag{A.8}$$

where s_1 is the focal sentiment, s_2 the sentiment of the other agent, ϵ the sentiment difference at which the update is the largest, and α the strength of the update. The shape parameter η (fixed at 50) controls how well the smooth kernel approximated the discrete one. The linear alternative model updates latent and expressed sentiment based on the linear update rule,

$$L_\alpha(s_1, s_2) = s_1 + \alpha(s_2 - s_1). \tag{A.9}$$

The stateless alternative model removes the latent sentiment state u_i, updating expressed sentiment $\bar{e}$ directly based on the set of received and replied-to sentiment:

$$\bar{e}_i[t] \sim \widehat{\mathcal{N}}\left(\bigotimes_{e_k \in I_i[t-1,t]} B_{\alpha_i,\epsilon_i}(\bar{e}_i[t-1], e_k), \sigma_i\right). \tag{A.10}$$

SLEBC Parameter Inference

The posterior distribution of the parameters for the SLEBC model $(\epsilon, \alpha_u, \alpha_e, \sigma_u, \sigma_e)$ was obtained independently per user with the means of a Hamiltonian Monte Carlo method, with an exponential prior with mean 0.5 for each σ and each α, and a uniform prior on [0,2] for ϵ [51]. The latter prior was chosen such that each meaningful value of ϵ was equally likely. The prior for the α's was chosen to allow high values to capture strong updates, while still limiting the ability to cross the entire sentiment spectrum in a single interaction. Results comprised 3000 samples, generated by a no U-turn sampler in six independent chains, each initiated by 250 discarded warm-up samples [52]

Watanabe-Akaike Information Criterion (Section 2.5)

The SLEBC model fit was quantified using the Watanabe-Akaike Information Criterion [53]. Given M samples θ_m of the posterior distribution containing model parameters $(\epsilon, \alpha_u, \alpha_e, \sigma_u, \sigma_e)$ and N observed sentiments s_j, the log pointwise predictive density is

$$\mathrm{lppd} = \sum_{j=1}^{N} \log\left(\frac{1}{M}\sum_{m=1}^{M} p\left(s_j \middle| \theta_m\right)\right) \tag{A.11}$$

This log likelihood increases if the sampled parameters better predict the observed sentiment. A penalty term, the effective number of parameters n_{eff}, is added to account for model complexity, calculated as the sum of the variances of the log likelihood across posterior samples,

$$n_{eff} = \sum_{j=1}^{N} \mathrm{Var}_{m=1}^{M}(\log p(s_j|\theta_m)) \ . \tag{A.10}$$

From these two values, the WAIC can be calculated as

$$WAIC = -2(\mathrm{lppd} - n_{eff}) \tag{A.11}$$
