# Supplementary material for: Social Contagion in COVID-19 Discussions Within the Belgian Reddit Community: Statistical and Modeling Study
Source: J Med Internet Res. 2026 Jul 29;28:e87723. doi: 10.2196/87723 (PMC13419282; doi:10.2196/87723)
Supplement: Multimedia Appendix 4 [file jmir-v28-e87723-s004.pdf]

Sensitivity Analysis of the Homophily Measure

As the homophily measure, h depends on the bin width w_H, we conducted a sensitivity analysis by varying w_H and observing the effects on h. We calculated h for five different values of w_H, as well as its relative difference with the smallest one,

$$\delta h(w_H) = \frac{h|_{w_H} - h|_{0.00125}}{h|_{0.00125}}.$$

Table A.1 presents the results of this analysis. While the effect of varying w_H depends on the topic, we conclude that $w_H = 0.05$ is an appropriate choice as it provides a good scale to interpret the figures and does not differ more than 0.2% from the smallest tested bin width.

	Lockdowns		*Masks*		*Vaccination*	
w_H	$h\|w_H$	δh	$h\|w_H$	δh	$h\|w_H$	δh
0.2	0.24468	-0.00626	0.21223	-0.00052	0.14548	0.01298
0.1	0.24571	-0.00209	0.21232	-0.0001	0.1446	0.00686
0.05	0.24614	-0.00033	0.21205	-0.00138	0.14387	0.00181
0.025	0.24586	-0.00146	0.212	-0.00157	0.14304	-0.00401
0.00125	0.24622	0	0.21234	0	0.14361	0

Table A.1: Sensitivity analysis of homophily measure h with respect to input bin width w_H.
